# Bindehautpigmentierung – Tumor oder Trauma?

**DOI:** 10.1007/s00347-021-01337-0

**Published:** 2021-02-25

**Authors:** Annika Müller-Kassner, Tschingis Arad, Ingo Schmack, Thomas Kohnen

**Affiliations:** Klinik für Augenheilkunde, Goethe-Universität Frankfurt, Theodor-Stern-Kai 7, 60590 Frankfurt am Main, Deutschland

## Anamnese

Eine 67-jährige Patientin wurde zur Mitbeurteilung bei unklarer Bindehautpigmentierung am linken Auge überwiesen. Bei einer augenärztlichen Routineuntersuchung seien erstmalig eine fokale Bindehautpigmentierung sowie eine periphere vordere Synechierung verbunden mit Defekten der Iris aufgefallen.

Anamnestisch sei der Patientin vor einigen Monaten diese Hyperpigmentierung der Bindehaut aufgefallen. Auf Nachfrage berichtete sie von einem Fahrradsturz vor 6 Monaten. Subjektiv sei sie beschwerdefrei.

In der Vorgeschichte fand sich eine Kataraktoperation, die 6 Jahre (rechts) bzw. 10 Monate (links) zuvor komplikationslos auf beiden Augen durchgeführt worden sei. Ferner sei am rechten Auge zusätzlich eine Yttrium-Aluminium-Granat(YAG)-Kapsulotomie erfolgt. Ophthalmologische Erkrankungen wurden verneint.

## Befund

**Figure 1:**
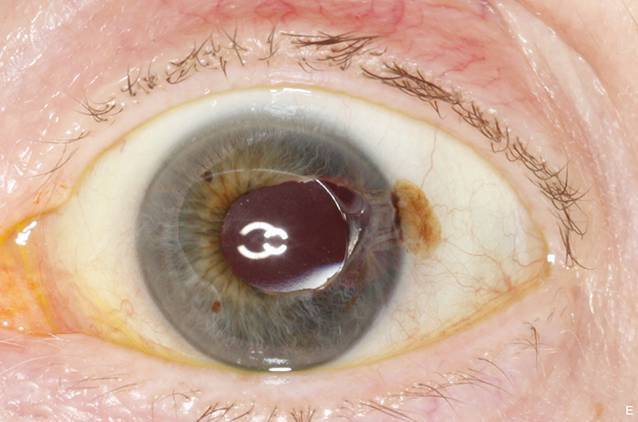

Bei Aufnahme betrug der bestkorrigierte Visus am linken Auge 0,8. Stereobiomikroskopisch zeigte sich am linken Auge eine temporale fokale Bindehautpigmentierung, bedingt durch einen subkonjunktivalen Irisprolaps bei 3 Uhr mit begleitender Verziehung und Ausdünnung des angrenzenden Irisgewebes (Abb. 1). Der Seidel-Test war schwach positiv. Die Intraokularlinse befand sich im Kapselsack. Fundoskopisch zeigte sich ein regelrechter Befund mit allseits anliegender Netzhaut.

## Wie lautet Ihre Diagnose?

## Therapie und Verlauf

Obwohl anzunehmen ist, dass der Irisprolaps im Bereich des ehemaligen Hauptschnitts (Clear-corneal-Inzision) als Folge einer Kontusion bei anamnestischem Sturzereignis der Patientin aufgetreten ist, lässt sich diese Vermutung im Nachhinein nicht zweifelsfrei belegen. Aufgrund der potenziellen Infektionsgefahr bei persistierender Kommunikation zwischen Vorderkammer und Augenoberfläche bzw. Außenluft (positiver Seidel-Test) wurde entschieden, den Irisprolaps operativ zu revidieren. Vorübergehend wurden zunächst eine lokale Antibiose und eine harte Klappe verordnet. Die Wundversorgung erfolgte stationär in Peribulbäranästhesie.

**Figure 2:**
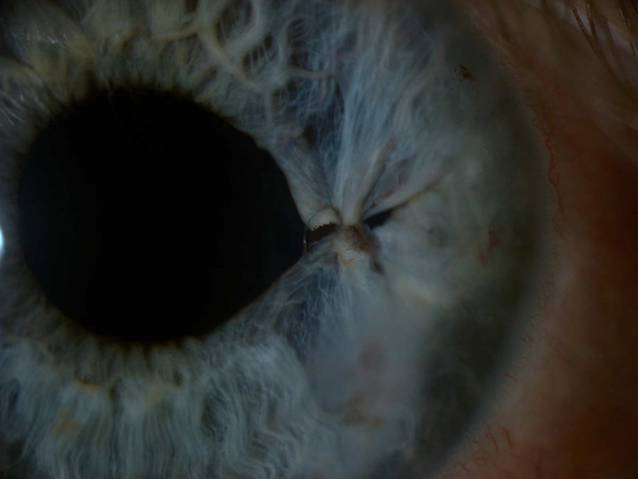

Eine beabsichtigte Reponierung des prolabierten Irisgewebes war aufgrund einer kompletten Adhäsion mit dem stromalen Hornhautgewebe sowie einer Atonie des Irisgewebes nur partiell möglich. Das übrige Irisgewebe wurde schonend abgetragen. Infolge einer vermehrten intraoperativen Blutung aus dem Irisstroma war intraoperativ wiederholt der Einsatz von dispersiven und kohäsiven Viskoelastika erforderlich. Zur Pupillenrekonstruktion wurde der entstandene Gewebsdefekt mit einer Irisnaht verschlossen und zusätzlich der Hornhauttunnel vernäht (Abb. 2 und 3).

**Figure 3:**
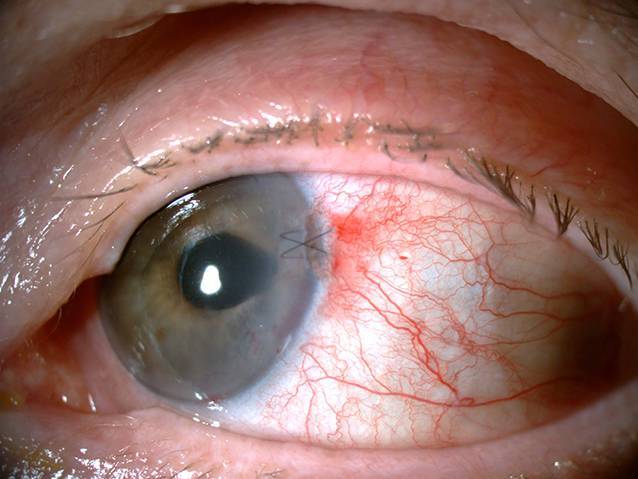

Postoperativ bestanden vorübergehend eine geringfügige Keratopathie im Wundbereich mit Ausbildung eines Hornhautstromaödems sowie eine Vorderkammereinblutung. Beides zeigte sich während des stationären Aufenthalts rasch rückläufig. Bei Entlassung betrug der korrigierte Visus am linken Auge 0,4.

Bei einer Verlaufskontrolle 3 Wochen postoperativ zeigte sich ein stabiler Befund mit einem bestkorrigierten Visus von 0,63. Die Fadenentfernung des Hornhautfadens ist in ca. 2 bis 3 Monaten geplant.

## Diskussion

Seit den frühen 1990er-Jahren wird in der minimal-invasiven Kataraktchirurgie der korneale Zugang am häufigsten mittels Clear-corneal-Inzision (CCI) durchgeführt [3]. Neben einer kürzeren Operationszeit, geringerer Blutungsrate und verminderter Astigmatismusinduktion zeichnet sie sich durch eine schnellere visuelle Rehabilitation als bei den skleral angelegten Schnitten (Corneal-scleral-Inzisionen [CSI]) aus [1].

**Diagnose:** Linkes Auge: transkornealer Irisprolaps bei Zustand nach Kataraktoperation

Bei der vorgestellten Patientin lag ein traumatischer Irisprolaps entlang der zuvor vorgenommenen kornealen Inzision mit partieller Wundtamponade durch das Irisgewebe vor.

Jegliche chirurgischen Zugänge am Auge stellen einen postoperativen Locus minoris resistentiae dar. Diese iatrogenen Schwachstellen begünstigen das Auftreten postoperativer Komplikationen. Stonecipher et al. wiesen ein erhöhtes Risiko von Wunddehiszenzen bei kornealen CCI im Vergleich zur skleralen CSI nach [6].

Es existieren Fallbeschreibungen, in denen von Wunddehiszenzen bis zu 6 Jahre postoperativ berichtet wird [5]. Gegenüber unserer Patientin bestand hierbei in den meisten Fällen eine Breite der CCI von > 3 mm [4, 7]. Insgesamt scheint die Geometrie des Schnittes einen entscheidenden Einfluss auf die postoperative Wundfestigkeit zu haben. So konnte anhand von Untersuchungen gezeigt werden, dass seitengleiche, quadratische Inzisionen stabiler sind als Inzisionen mit verschieden langen Seiten (zu kurze oder lange Wundtunnel) [2].

Zur Prävention von traumatischen Wunddehiszenzen nach CCI sollte demnach auf die korrekte Anlage der Inzisionen mit einer möglichst geringen Breite und einer quadratischen Konfiguration sowie einer ausreichenden stromalen Hydratation nach Implantation der IOL geachtet werden. Bei Bedarf kann zudem ein Nahtverschluss der Inzision erwogen werden.

Zusammenfassend stellt die CCI eine sichere Methode mit deutlichen Vorteilen hinsichtlich des funktionellen postoperativen Ergebnisses dar. Nichtsdestotrotz repräsentiert die korneale Wunddehiszenz mit Prolaps von intraokularen Strukturen eine mögliche Komplikation sowohl in der frühen als auch späten postoperativen Phase.

## Fazit für die Praxis

- Korneale Inzisionen können über Monate bis Jahre persistierende mechanische Schwachstellen darstellen.
- Die korrekte Schnittführung bei der Generierung kornealer Inzisionen ist zur Vermeidung von postoperativen Wunddehiszenzen und der Gefahr des Auftretens von Endophthalmitiden maßgeblich.
